# The verb–self link: An implicit association test study

**DOI:** 10.3758/s13423-022-02105-0

**Published:** 2022-05-02

**Authors:** Patrick P. Weis, Jan Nikadon, Cornelia Herbert, Magdalena Formanowicz

**Affiliations:** 1 Department of Psychology, Center for Research on Social Relations, SWPS University of Social Sciences and Humanities, ul. Chodakowska 19/31, 03-815 Warsaw, Poland; 2 Applied Emotion and Motivation Psychology, Institute of Psychology, Ulm University, Ulm, Germany; 3 Present Address: Department of Psychology (III), Julius Maximilian University, Röntgenring 11, 97070 Würzburg, Germany

**Keywords:** Language, Verbs, Self, Agency

## Abstract

Agency is defined as the ability to assign and pursue goals. Given people’s focus on achieving their own goals, agency has been found to be strongly linked to the self. In two studies (*N* = 168), we examined whether this self–agency link is visible from a linguistic perspective. As the preferred grammatical category to convey agency is verbs, we hypothesize that, in the Implicit Association Test (IAT), verbs (vs. nouns) would be associated more strongly with the self (vs. others). Our results confirmed this hypothesis. Participants exhibited particularly fast responses when reading self-related stimuli (e.g., “me” or “my”) and verb stimuli (e.g., “deflect” or “contemplate”) both necessitated pressing an identical rather than different response keys in the IAT (*d* = .25). The finding connects two streams of literature—on the link between agency and verbs and on the link between self and agency—suggesting a triad between self, agency, and verbs. We argue that this verb–self link (1) opens up new perspectives for understanding linguistic expressions of agency and (2) expands our understanding of how word choice impacts socio-cognitive processing.

## General introduction

Humans are vigilant toward cues of agency, defined as the ability to assign goals and plan and execute their achievement (Bakan, 1966; ). We pay attention to signals of animacy (Guerrero & Calvillo, 2016), biological motion (Simion et al., 2008), causality, and intentionality (Frith & Frith, 2010), and we do this because people who show these properties may act upon their goals and thus collide or align with our own goal-oriented activities (New et al., 2007). Accordingly, sensitivity to cues of agency is specifically evident in contexts that activate one’s goal orientation (Cislak, 2013).

Given that one’s own goals are highly accessible to an individual and that we use the self as a prototypical agent when attempting to monitor and understand goal-directed activity in general (Bornkessel & Schlesewsky, 2006; Dahl, 2008), our own agency is more relevant to us than the agency of others (Abele & Wojciszke, 2007, 2014). In the present study, we examine linguistic manifestations of this phenomenon—that is, whether self-related words (here, pronouns; e.g., “my” and “me”) have a stronger connection to linguistic expressions of agency (here, verbs; Formanowicz et al., 2017) than other-related words (here, pronouns; e.g., “his” or “him”). To examine this hypothesis, we applied the Implicit Association Test (IAT; Greenwald et al., 1998), a task frequently used to measure mental relations between concepts.

### Agency and the self

Given people’s focus on achieving their goals, it is not surprising that a plethora of research linked agency to the self (Abele & Wojciszke, 2007, 2014). For example, when participants rated various personality traits on their agency (i.e., how much they refer to goal orientation and how much they serve self-interest), the correlation was high (*r* = .69; Abele & Wojciszke, 2007, Study 1). When asked to describe themselves, participants provided more agentic traits than when describing an acquaintance (Bruckmüller & Abele, 2013, Study 1). Furthermore, when given an option of a self-enhancement workshop for oneself or for other people, participants preferred a workshop that increased agency for themselves, while for others they suggested a workshop focusing on the development of relationship skills (Abele & Wojciszke, 2007, Study 3). Given the importance of agency for the self, also not surprisingly, perceptions of one’s agency are also the strongest predictor of self-esteem evaluations (Abele & Hauke, 2018; Wojciszke et al., 2011). Altogether, studies indicate a strong connection between the concepts of self and agency.

These findings in the social domain are in accord with research in psycholinguistics. There is a basic tendency to differentiate between the categories of agent and patient as evident from studies of linguistic topology, newly emerging sign languages, and language comprehension (for a review, see Rissman & Majid, 2019). The extended argument dependency model for language processing (Alday et al., 2014; Bornkessel & Schlesewsky, 2006) specifies that sentence comprehension follows the same general tendency to detect cues of agency as precisely and quickly as possible (Frith & Frith, 2010) because it is crucial to determine who plays the role of an agent. Accordingly, across languages, the position of agent (i.e., the one responsible for what is happening) is a privileged position for sentence processing (Gardelle & Sorlin, 2018).

In a similar vein, agents can be identified in reference to agent prototypicality, with some agents being more prototypical and thus more probable in that role (see also below for linguistic prominence). Due to the preference for the self as a prototypical agent among animate targets, the self is the most likely agent candidate and serves as a benchmark for understanding and interpreting other people’s actions (Bornkessel & Schlesewsky, 2006). Overall, findings from the domains of both psycholinguistics and social psychology point to the privileged connection of self and agency (cf. Dahl, 2008; Gardelle & Sorlin, 2018).

### Verbs and agency

Following up on the linguistic presence of agency references, in terms of grammatical categories, verbs seem particularly suited to express content related to agency (Fausey & Boroditsky, 2011; Formanowicz et al., 2017). Verbs typically imply dynamic properties that other grammatical categories, such as nouns or adjectives, lack, making them the preferred syntactic devices to convey activity (Cappa & Pulvermüller, 2012; Foroni & Semin, 2009; Vigliocco et al., 2011) and, by extension, agency. For example, in a series of experiments, when participants rated pseudowords with the same word stem and different suffixes and assigned the word to the grammatical category of verb (e.g., to *frosh*), adjective (e.g., *froshive*), or noun (e.g., *froshness*), verbs were interpreted as more agentic than other grammatical categories (Formanowicz et al., 2017). Importantly, verbs were related only to agency but not to psycholinguistic dimensions of abstractness or valence nor to the dimension of communion, that is, an orientation toward a relationship with others (Abele & Wojciszke, 2007, 2014). Thus, the link between verbs and activity mostly affects the verbs’ relation to agency. Other grammatical categories seem to play a more minor role in the subtle transmission of agency information (Formanowicz et al., 2017).

The link between verbs and agency has also been confirmed in natural language use. When social categories that are stereotypically associated with high agency (e.g., men, young people) were compared with low-agency stereotypes (e.g., women, the elderly), the former were more often associated with a verb than the latter (Formanowicz et al., 2017; for a similar pattern of results in eye-tracking studies, see Esaulova & von Stockhausen, 2015). This relationship is akin to the concept of linguistic prominence, in which the likelihood of an argument occurring in the thematic role of an agent versus patient is also related to its animacy, as understood in terms of intentionality and autonomy (De Hoop & Lamers, 2006; Muralikrishnan et al., 2015; Vihman & Nelson, 2019). Importantly, the self is positioned as the most prototypical agent.

### Overview of the current research: Verbs, self, and agency

Building on these theoretical assumptions and the results of previous work, this study’s aim is to investigate the hypothesis that verbs, as linguistic markers of agency, are associated more strongly with linguistic markers that reference the self, such as first-person personal pronouns than personal pronouns that reference others. Furthermore, if verbs are strong primers of agency addressing the self, we expect the association between self and verbs to also be stronger than the link between self and a grammatical category that is more neutral in respect to agency (i.e., nouns). To test these hypotheses, we conducted two experiments using customized versions of the IAT (Greenwald et al., 1998), a test that has already been successfully used to investigate the concept of the self (Greenwald & Farnham, 2000).

## Experiment 1

**Fig. 1:**
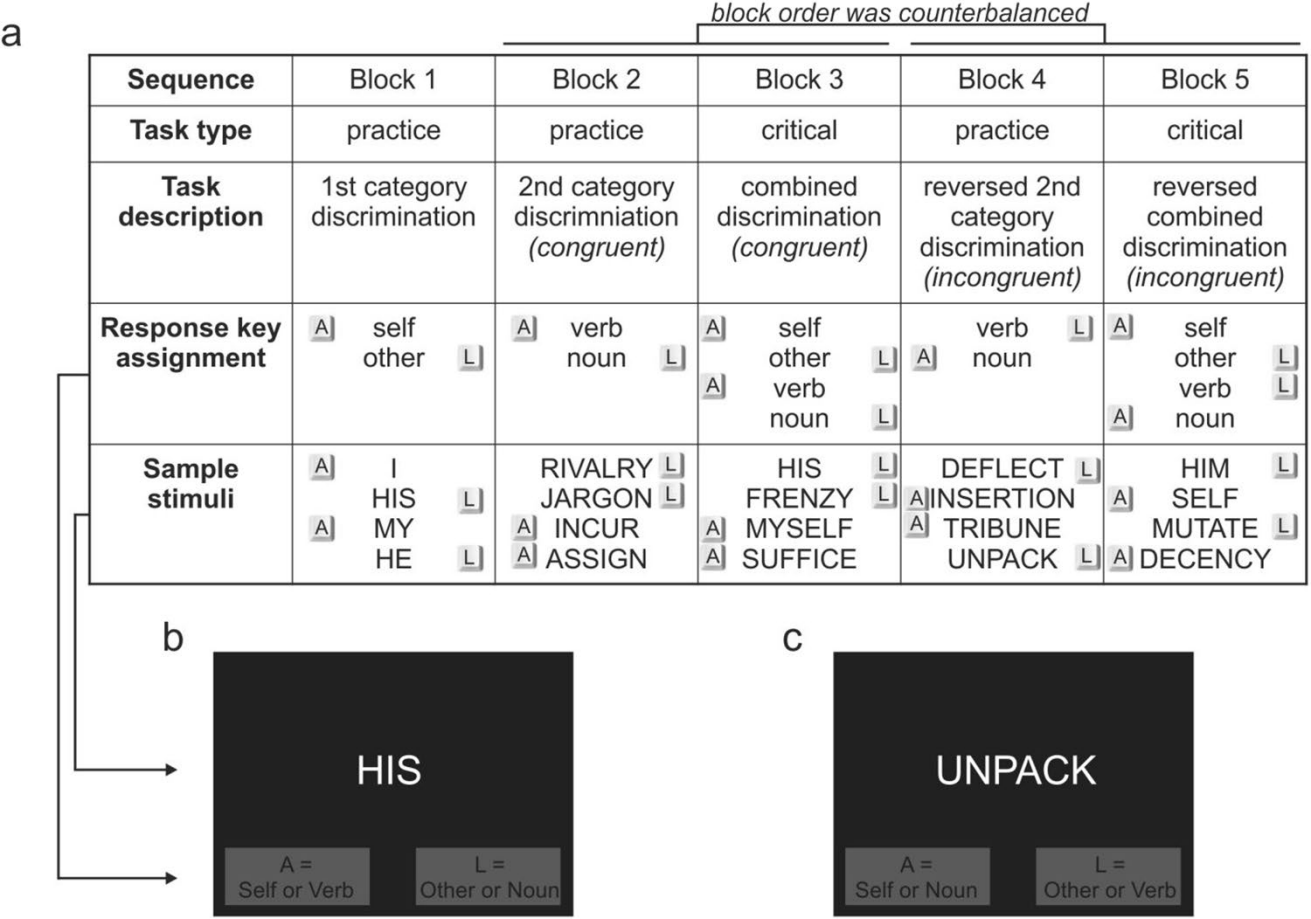
IAT design. *Note*. The main experiment consisted of five blocks (**a**). The key caps drawn in the “Response key assignment” row indicate the assignment of category labels to response keys. In other words, the “A” and “L” key caps indicate whether a category label was written on the left side and paired with the “A” key or on the right side and paired with the “L” key. The key caps in the “Sample stimuli” row indicate correct key presses for sample stimuli. To illustrate the response key assignments, a congruent Block 3 example trial (**b**) and an incongruent Block 5 example trial (**c**) are given. Stimuli are not drawn to scale. Note that verb and noun stimuli were carefully matched for valence and other dimensions (see Table 1). To balance order effects, the order was counterbalanced. For half of the participants, blocks were presented in the order shown in (**a**), i.e., 1, 2, 3, 4, 5; for the other half, blocks were presented in an altered order (i.e., 1, 4, 5, 2, 3). Less importantly, the response key assignment was also counterbalanced so that, for half of the participants, the key assignment was the opposite of what is depicted in (**a**). For further details, see the IAT Task section

Experiment 1 was the first attempt to test the hypothesis that verbs are associated more closely with the self than with others. Participants had to categorize either nouns and verbs (e.g., participants read “deflect” and needed to answer “verb”) or self- and other-related pronouns (e.g., participants read “his” and needed to answer “other”). Trials in which participants had to press an identical key for verbs and for self-related pronouns were labelled *congruent* trials. Trials in which participants had to press a different key for verbs and for self-related pronouns (i.e., when verbs and other-related pronouns share a key) were consequently labelled *incongruent* trials—for details on IAT procedure, see Fig. 1. The resulting IAT effect measured how much faster participants answered congruent versus incongruent trials. The IAT effect thus reflects the associative strength between verbs (which indicate high agency) and self-related pronouns (which indicate relevance to the self). Our expectation was that the IAT effect is positive and significantly different than zero. In other words, we expected congruent trials to be answered faster than incongruent trials.

### Methods

#### Participants

An a priori power analysis conducted in G*Power (Faul et al., 2007) indicated that 84 participants would be needed to find an effect with a small to medium size (*f* = 0.2, 1 − *β* = 0.95, correlation between repeated measurements = 0.5). We continued recruiting participants until our sample size matched the power analysis after all exclusion criteria had been applied. Participants were recruited from the United States, Canada, and the United Kingdom through the platform Amazon Mechanical Turk (www.mturk.com). Participants with both a failed attention check (“What is the current year?”) and an IAT categorization accuracy of below 60% (see IAT Task section) did not enter further analysis because the data were likely either produced by highly inattentive individuals or computer programs (cf. Fleischer et al., 2015). In accordance with Greenwald and Farnham (2000) and to avoid distorting IAT effects due to inattentive participants or participants with insufficient English language skills, we additionally excluded 32 participants due to a categorization accuracy of below 80%, which resulted in a sample of 84 participants (40 female, 43 male, one participant who preferred not to disclose their gender; *M*_age_ = 45.4, *SD*_age_ = 12.1, range_:_ 24–73 years).

#### Apparatus

Participants completed the task on their personal computer running either Windows or MacOS. The task was programmed using PsychoPy and PsychoJS (Version 2020.2; see Peirce et al., 2019) and presented online via the Pavlovia platform (www.pavlovia.org).

#### Procedure

After clicking the link to the experiment provided at Mechanical Turk and providing informed consent, participants were welcomed to the study and introduced to the IAT procedure. The present IAT investigated associations between Personal Reference and Word categories (see Table 1) and is described in more detail in the IAT Task section. After completing 220 IAT trials, participants were asked to fill out several questionnaires. First, participants answered one item inspired by the Inclusion of Other in the Self (IOS) scale (“Which picture best describes your relationship with the persons you had in mind when reading ‘HE,’ ‘HIS,’ ‘HIM,’ ‘HIMSELF,’ or ‘OTHER’?”; Aron et al., 1992). Answers ranged from 1 (*no overlap between self and other*) to 7 (*very high overlap between self and other*). The IOS scale was conducted to explore whether the present IAT effect measure might diminish with increasing incorporation of the other person into the self. Second, participants answered 20 items that captured the participants’ agency and communion (Agency and Communion Questionnaire [ACQ]; Abele et al., 2016) on a 5-point scale. For example, participants were asked to “indicate how much the following applies to you: capable,” from “little capable” to “very capable.” We speculated that the high agency conveyed by verbs would render verbs more closely associated with the self. Thus, the ACQ was conducted to explore whether the present IAT effect measure would scale with a person’s agency. Third, to check the composition of our sample, participants answered six questions about themselves (e.g., gender and education). Lastly, participants rated all target words with respect to their agency. Similar to the procedure employed by Formanowicz et al. (2017), agency was introduced as “the orientation toward actions and being efficient. It is about striving to achieve goals. For example, the words activity, success, strive, or achieve are usually rated to have very high agency” (p. 6), and participants were asked to rate the words on a five-point scale ranging from “no agency” to “very high agency.” Agency ratings were implemented to check whether nouns and verbs differed in agency despite the fact that we carefully matched verbs and nouns on a wide range of parameters. If we found a difference in agency, this would strengthen the findings from earlier research (Formanowicz et al., 2017) that agency differences are inherently linked to differences in grammatical categories (see Language Stimuli section for details). After completing the ratings, participants were provided with a code to get reimbursed, thanked, and wished farewell.

**Table 1:** Word category stimuli for Experiment 1

Matched pair		Arousal		Concreteness		Dominance		Valence		Frequency		Letters	
*noun (n)*	*verb (v)*	*n*	*v*	*n*	*v*	*n*	*v*	*n*	*v*	*n*	*v*	*n*	*v*
cruiser	unpack	2.9	2.9	3.78	3.82	5.7	5.45	5.32	5.29	5	5	7	6
vacancy	deflect	3.68	3.68	3.28	3.25	5.21	5.37	5.05	5.1	7	5	7	7
tribune	assign	3.78	3.71	2.93	2.86	5.71	5.74	4.84	4.75	8	19	7	6
insertion	infiltrate	5.05	5.13	2.56	2.71	5.18	5.11	4.47	4.41	1	4	9	10
frenzy	mutate	5.96	5.88	2.31	2.52	4	4.04	4.2	4.16	7	1	6	6
rivalry	vanquish	5.05	5.05	2.14	2.21	5.64	5.29	4.57	4.72	8	2	7	8
jargon	incur	3.95	4	2.07	2	5.25	5.04	4.85	4.8	7	6	6	5
decency	suffice	3.36	3.25	2	1.72	6.21	6.19	5.55	5.52	5	6	7	7
theology	reinstate	3.32	3.33	1.93	1.9	5.35	5.25	4.95	5.11	8	2	8	9
likelihood	contemplate	2.9	3.16	1.73	1.71	5.59	5.68	5.74	5.48	11	18	10	11
		4.00	4.01	2.47	2.47	5.38	5.32	4.95	4.93	6.70^a^	6.80^b^	7.4	7.5

Arousal, dominance, and valence were rated on a 9-point scale with a midpoint of 5. Concreteness was rated on a 5-point scale with a midpoint of 3. Frequency refers to the total word frequency out of a million words, and letters refer to the number of letters. For further details on the word selection procedure, see the Language Stimuli section. The bottom row represents the column means

^a^The corresponding logarithmic value is log(6.70) = 1.90

^b^The corresponding logarithmic value is log(6.80) = 1.92

#### IAT task

An IAT enables measuring the association between categories (Greenwald et al., 1998). The design used for the present study follows the typical experimental block design, as used by Greenwald et al. (1998). This particular IAT was designed to investigate associations between Personal Reference and Word categories. In Block 1, participants completed 20 practice trials in which they were asked to categorize pronouns as belonging to either the “self“ or to the “other” Personal Reference Category. In Block 2, participants then categorized 20 words as belonging to the “verb” or to the “noun” word category. Block 3 consisted of 80 combined discrimination trials, during which participants categorized words from the Personal Reference and Word categories at the same time. In Block 4, participants then engaged in 20 Word categorization practice trials, in which the key assignment was reversed compared with the previous trials. Lastly, in Block 5, participants engaged in 80 combined categorization trials, again with the reversed key assignment compared with the word category. Each participant thus completed 60 practice and 160 critical trials, for a total of 220. The IAT procedure is summarized in Fig. 1.

To allow comparison with previous studies, the present IAT closely mirrors a previously designed IAT that also used self- and other-related pronouns (Greenwald & Farnham, 2000). Except for the second category (i.e., the word category, consisting of nouns and verbs), there are two noteworthy differences. The first difference is that the present experiment uses 80 combined and 80 reversed combined trials instead of 60 combined and 60 reversed combined trials. This change was necessary to balance the amount of word category stimuli (i.e., 20) and the amount of Personal Reference stimuli (i.e., 10) so that eventually participants could engage in 40 trials each. The second difference is that, in addition to counterbalancing whether combined or reversed combined discrimination was encountered first (i.e., block sequence was 1, 2, 3, 4, 5 for half the participants and 1, 4, 5, 2, 3 for the other half), we also counterbalanced whether “self” was assigned to the left (“A” key) or to the right (“L” key).

All word stimuli (for details, see Language Stimuli section) were preceded by a fixation cross for 150 ms and were presented until either the “A” or “L” key was pressed to categorize the target word. No immediate error feedback was provided. Instead, the mean response time and error rate in percent was shown after each block to encourage participants to respond as quickly and accurately as possible. During critical trials (i.e., Blocks 3 and 5), stimulus items were drawn alternately from the Personal Reference list (odd-numbered trials) and the word category list (even-numbered trials), a procedure in accordance with former IAT studies (Greenwald et al., 1998; Greenwald & Farnham, 2000). Words from each list were selected randomly and without replacement so that all words were used once before any words were reused.

#### Language stimuli

For Personal Reference, 10 words were used: I, ME, MY, SELF, and MYSELF for self-reference and HE, HIS, HIM, OTHER, and HIMSELF for other-reference (for a set of similar IAT pronoun stimuli, consult Greenwald & Farnham, 2000).

For the word category, we carefully matched 10 nouns with 10 verbs based on an evaluation of several parameters. To create a comparable set of *nouns* and *verbs,* we used ratings of valence, arousal, and dominance (measured on 9-point scales with end points unhappy/happy, calm/excited, and controlled/in control, respectively; Warriner et al., 2013), concreteness (5-point scale; Brysbaert et al., 2014), frequency (absolute word frequency out of a million, as extracted from the CELEX lemma database; Baayen et al., 1995), and length in letters.

Before matching nouns and verbs, we filtered words with nonneutral valence (below 4 or above 6). Using the remaining words, we matched nouns and verbs based on four parameters: arousal, concreteness, dominance, and valence. None of a pair’s matched parameter values deviated more than 0.4. Additionally, no verb was allowed to deviate more than one letter in word length from the matched noun. Out of the resulting pairs, we picked the ones that had the most similar average word frequency. Pairs and parameters can be inspected in Table 1.

#### Data analysis

The analytic procedure followed recommendations made by Greenwald et al. (2003) and was based on 80 combined and 80 reversed combined discrimination trials per participant. In line with Greenwald et al. (2003), practice blocks were included in the analyses to increase power; participants with more than 10% of trials with latencies below 300 ms were excluded (0 participants); all trials with latencies below 400 (0.15% of all trials) and above 10,000 ms (0.03% of all trials) were excluded from the analysis; and error trial latencies were replaced with mean latencies of the correct trials in the respective block plus 600 ms (8.7% of all trials). Computation of standardized reaction time (RT) differences between combined discrimination and reversed combined discrimination blocks also followed recommendations from Greenwald et al. (Table 4 in Greenwald et al., 2003). In short, the computation of standardized RT differences entailed computing the RT difference between congruent and incongruent trials and dividing it by the pooled standard deviation of the respective individual.

The IAT effect was analyzed with a one-sample *t*-test comparing the standardized RT differences (between congruent and incongruent blocks) against zero. We also used a multilevel modeling approach with two random effects—intercepts for both participants and word stimuli—to validate the IAT effect while controlling for the random variance induced by the specific words we selected. Congruency (congruent: self/verb on one key and other/noun on the other; incongruent: self/noun on one key and other/verb on the other) was entered as an independent variable. The random coefficient model used raw rather than standardized reaction times because the common standardization procedure for the IAT (Greenwald et al., 2003) results in only one standardized value per participant and, therefore, prohibits item-wise analysis. The model was implemented using R software (Version 3.6.3; R Core Team, 2013) and the lme4 package’s function lmer (Version 1.1-19; Bates et al., 2015). Marginal means were computed using the emmeans^1^ package.

### Results^2^

#### IAT effect

**Fig. 2:**
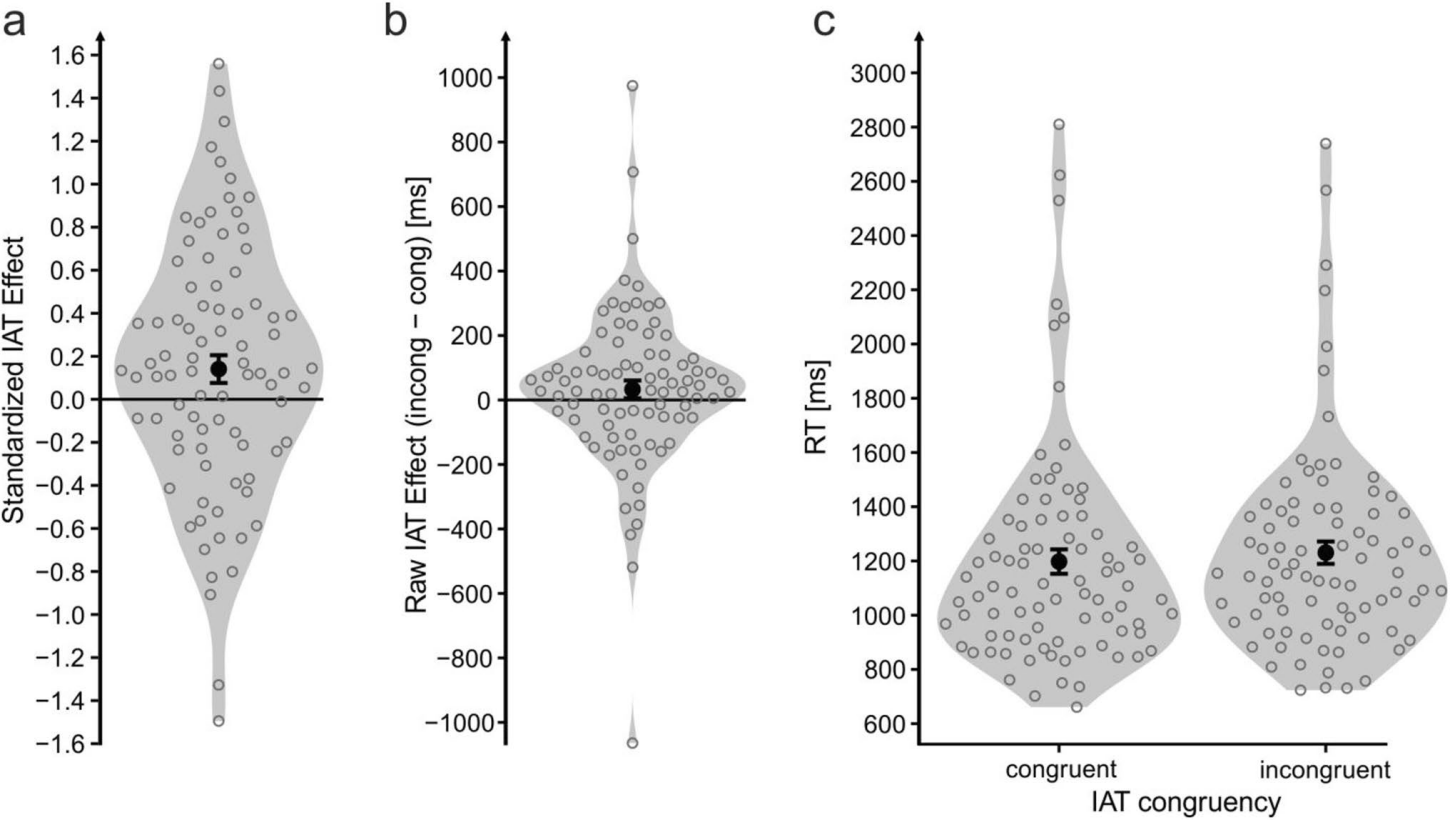
IAT effect in Experiment 1. *Note*. Analysis is based on the standardized Implicit Association Test (IAT) effect (**a**). For data transparency reasons, the raw IAT effect (**b**) as well as the raw reaction time data (**c**) is illustrated as well. Large black dots indicate the average of the whole sample. Black error bars indicate the standard error of the mean. Gray circles indicate the average of a single individual. Plotting individual data points is in line with statistical recommendations (e.g., Cumming, 2013). Gray shapes represent violin plots, as implemented by ggplot2 (Wickham, 2016). Note that the within-participants variance relevant for the present analysis cannot be inferred from (**c**)

Participants exhibited an IAT effect in the hypothesized direction. Participants answered faster in congruent compared with incongruent trials (*M*_Δ_ = .140), *t*(83) = 2.18^3^, *p* = .032, *d* = 0.24 (Fig. 2a). Note that this analysis was performed using standardized IAT effect scores (Greenwald et al., 2003). For data transparency reasons, the unstandardized IAT effect (*M*_Δ_ = 33 ms; Fig. 2b) and raw RTs (*M*_congruent_ = 1,198 ms, *M*_incongruent_ = 1,231 ms; Fig. 2c) are illustrated as well. The IAT effect was validated using the multilevel modeling approach described in the Data Analysis section (*M*_Δ_ = 33 ms), *t*(13308) = 3.11, *p* = .002 (for model details, see Table 2). Accuracy was higher in congruent (*M*_congruent_ = .93) than in incongruent (*M*_incongruent_ = .92), *t*(83) = 2.64, *p* = .001, trials.

**Table 2:** Multilevel model results for the unstandardized Implicit Association Test (IAT) effect in Experiment 1

Unstandardized IAT effect[s]				
Random Effects	Variance	*SD*		
Participants	0.14	0.37		
Words	0.05	0.21		
Residual	0.37	0.61		
Fixed effects	Estimate	*SE*	|*t*|	
Intercept	1.253	0.06	22.08	
Congruency	0.033	0.01	3.11	

Congruency was comprised of two levels (congruent: self/verb on one key and other/noun on the other; incongruent: self/noun on one key and other/verb on the other)

A 2 × 2 exploratory analysis of variance (ANOVA) with the standardized IAT effect as the dependent variable and the key order (verb assigned to the “A” key and noun assigned to the “L” key or vice versa) and category matching order as independent variables revealed a main effect of the category matching order, *F*(1, 80) = 9.3, *p* = .003, η_G_^2^ = .10. That is, the IAT effect was larger when verb/self and noun/other were matched first during Blocks 2 and 3 (*M*_marginal_ = .299) than when verb/other and noun/self were matched first during Blocks 2 and 3 (*M*_marginal_ = −.093). Neither the key order, *F*(1, 80) = 1.2, *p* = .277, η_G_^2^ = .01, nor the interaction effect, *F*(1, 80) = .55, *p* = .461, η_G_^2^ < .01, were significant.

#### Inclusion of Other in the Self (IOS) Scale

The IOS score did not correlate with the standardized IAT effect (*r =* .002)*, t*(82) = 0.02, *p* = .99.

#### Agency and Communion Questionnaire (ACQ)

None of the ACQ subscales (AA, AC, CM, or CW) correlated with the standardized IAT effect, all |*t*|(81) < 1.40, all *p*s > .15, all *r*s between 0 and −0.15. One participant was excluded because of technical issues.

#### Agency ratings

Participants rated the verbs higher in agency than the nouns, as indicated by a two-sided dependent *t* test (*M*_Δ_ = 0.67), *t*(83) = 8.40, *p* < .0001, *M*_verbs_ = 3.16, *M*_nouns_ = 2.49.

### Discussion

Results from Experiment 1 confirm our hypothesis: The mean IAT effect was positive and significantly differed from zero. Self (vs. other) pronouns were more closely related to verbs than to nouns. However, exploratory analyses did not support the notion that the IAT effect is diminished when the concept of the other overlaps with the self. Exploratory analyses also did not support the notion that the IAT effect scales with trait agency or communion. To examine whether the results of Study 1 could have been influenced by the fact that the used stimuli have different co-occurrences in natural language use we also analyzed the contextual similarity of the stimuli (verbs and nouns vs. self- and other-related pronouns). We quantified the contextual similarity of the Experiment 1 stimuli using the cosine similarity measure applied directly to their vector space representations (embeddings) extracted from the English variant of the subs2vec embedded computational language model (Van Paridon & Thompson, 2021). Embedded language models are constructed by assigning vectors to words in such a way that the geometry of these vectors captures semantic relations between the words and specifically similar vector representations are assigned for words that occur in similar contexts (Van Paridon & Thompson, 2021). The details of this analysis are presented in the Supplemental Online Material (SOM). In sum, self-related (vs. other-related) pronouns occurred in similar contexts with respect to verbs. However, for self-related (vs. other-related) pronouns in relation to nouns, we observed differences in the natural language use. Specifically, self-related pronouns had lower contextual similarity to the nouns used in Experiment 1 than other-related pronouns. This could theoretically affect IAT results because participants may have linked nouns and self-related pronouns more slowly compared with nouns and other-related pronouns. We return to this issue in the General Discussion.

## Experiment 2

The IAT effect that was present in Experiment 1 suggests that verbs are closely associated with the self. To solidify this finding, we aimed at replicating Experiment 1 with the following methodological changes, while keeping everything else, including procedure and software, identical:

1. Limited sample of pronouns. In Experiment 1, “I” does grammatically pair with the infinitive of the verb (e.g., “I unpack”). However, “HE” does not pair well (e.g., “He unpack” would be grammatically incorrect). This possible confound might have upwardly biased the IAT effect. To exclude this possibility, both words were omitted. We also omitted the words MYSELF, HIMSELF, SELF, and OTHER because, otherwise, we would need to present some words more frequently than others in blocks consisting of 20 trials. Two words per category (here, MY, ME, and HIS, HIM) were shown to sufficiently induce solid IAT effects (Nosek et al., 2005).
2. Different verbs and nouns. The verb and noun stimuli used in Experiment 1 might not be generalizable. The IAT effect could, in principle, be driven by the specific verbs and nouns we selected rather than by their word category. This possibility is further bolstered by the fact that contextual similarity results indicate that self-related pronouns occurred in lower similarity with nouns used in Experiment 1 in comparison to other-related pronouns. Changing the stimuli list can allow us to examine whether contextual similarity of the pronouns used in Experiment 2 and different verbs and nouns can be linked to the results of the IAT. If the IAT effect persisted with a different set of verb and noun stimuli, this would strengthen the interpretation of word category as the underlying reason.
3. Different recruitment platform. In Experiment 1, we needed to exclude a large number of data sets with low task accuracy. This was at odds with similar experimental designs (e.g., Greenwald & Farnham, 2000) and not desirable because we did not know whether the low accuracy was produced by algorithms (“bots”), highly inattentive participants, or attentive participants who, for some reason, did not succeed in mastering the task at hand. A high proportion of inattentive participants or bots is undesirable because, by chance, some of these might score high accuracies and, if included in data analysis, would decrease the power and could bias the IAT effect estimate. Attentive but inaccurate participants are not desirable because this would indicate problems with the task. To exclude problems with our task and increase power, we therefore switched from Amazon Mechanical Turk to the participant recruitment service Prolific (www.prolific.co). Prolific employs fraudulence and participant pool checks that would enable us to collect data sets with higher accuracy due to fewer bots and more attentive participants (cf. Peer et al., 2017). If the IAT effect persisted in a sample with overall higher answer accuracy, this would strengthen the initial finding from Experiment 1 and allow for a more accurate IAT effect estimate.

Given these considerations, we hypothesized that the positive IAT effect from Experiment 1 can be replicated with different noun and verb stimuli, with a differently recruited sample, and without the possibly biasing pronouns I and HE.

### Methods

Except for the changes explicitly mentioned, the same procedure as in Experiment 1 was applied.

#### Participants

Participants were recruited from the United States, Canada, and the United Kingdom through the Prolific platform (www.prolific.co). Out of 108 collected data sets, 24 were excluded due to categorization accuracy below 80%, resulting in a final sample size of 84 (42 female, 41 male, 1 diverse; *M*_age_ = 35.4, *SD*_age_ = 13.8, range: 18–72 years). One participant did not enter their age.

#### Language stimuli

Stimuli were selected using the same criteria as in Experiment 1. The resulting word list can be seen in Table 3. As for Experiment 1, we also analyzed the contextual similarity of the experimental stimuli. We observed no statistically significant differences between stimuli (verbs and nouns vs. self-related and other-related pronouns) used in Experiment 2. For details on these analyses please consult the SOM.

**Table 3:** Word category stimuli for Experiment 2

Matched pair		Arousal		Concreteness		Dominance		Valence		Frequency		Letters	
*noun (n)*	*verb (v)*	*n*	*v*	*n*	*v*	*n*	*v*	*n*	*v*	*n*	*v*	*n*	*v*
icebreaker	cultivate	3.82	3.81	3.17	3.14	5.83	6.23	5.68	5.73	0	14	10	9
empire	operate	4.59	4.62	3	3	5.95	5.78	5.36	5.37	16	82	6	7
outreach	supervise	3.95	4	2.57	1	5.74	6.1	4.94	5.05	0	9	8	9
status	hasten	3.83	3.86	2.24	2.22	5.28	5.33	4.89	4.9	77	9	6	6
possession	circulate	4.04	4	2.96	2.88	5.47	5.68	5.14	5.19	45	13	10	9
loophole	confess	4.71	4.67	2.63	2.57	5.35	5.53	4.84	4.85	3	26	8	7
treaty	consult	3.7	3.77	3.07	3.04	5.62	5.83	5.42	5.41	19	32	6	7
reaction	regroup	3.95	4.09	2.41	2.41	6.3	6.28	4.68	4.7	73	2	8	7
origin	reduce	3.7	3.67	2.03	2	5.29	4.92	5.19	5.1	46	122	6	6
comparison	recollect	2.92	3.05	2	1.9	5.64	5.75	5.11	5.05	28	3	10	9
		3.92	3.95	2.61	2.56	5.65	5.74	5.13	5.14	30.7^a^	31.2^b^	7.8	7.6

Arousal, dominance, and valence were rated on a 9-point scale with a midpoint of 5. Concreteness was rated on a 5-point scale with a midpoint of 3. For further details on the word selection procedure, see the Language Stimuli section from Experiment 1. The bottom row represents the column means

^a^The corresponding logarithmic value is log(30.7) = 3.42

^b^The corresponding logarithmic value is log(31.2) = 3.44

### Results^4^

#### IAT effect

**Fig. 3:**
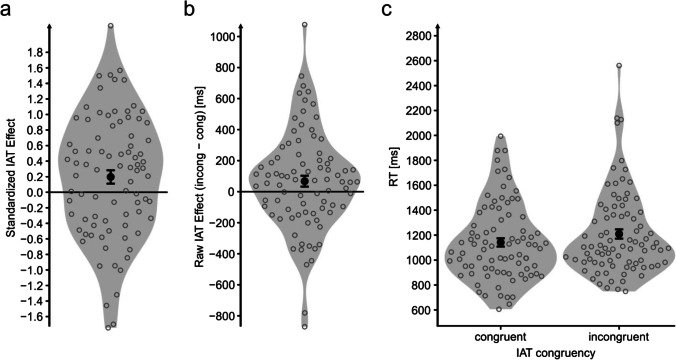
IAT effect in Experiment 2. *Note.* Analysis is based on the standardized Implicit Association Effect (IAT) effect (a). For data transparency reasons, the raw IAT effect (**b**) and the raw reaction time data (**c**) are illustrated as well. Large black dots indicate the average of the whole sample. Black error bars indicate standard error of the mean. Gray circles indicate the average of a single individual. Gray shapes represent violin plots as implemented by ggplot2 (Wickham, 2016)

Participants exhibited an IAT effect in the hypothesized direction. As indicated by a one-sided dependent *t* test, participants answered faster in congruent (i.e., self/verb on one key and other/noun on the other) compared with incongruent trials (*M*_Δ_ = .197), *t*(83) = 2.29^5^, *p* = .025, *d* = 0.25 (Fig. 3a). For data transparency reasons, the unstandardized IAT effect (*M*_Δ_ = 68 ms; Fig. 3b) and the raw RTs (*M*_congruent_ = 1,142 ms, *M*_incongruent_ = 1,209 ms; Fig. 3c) are illustrated as well. The IAT effect was validated using the multilevel modeling approach described in the Data Analysis section (*M*_Δ_ = 68 ms), *t*(13309) = 6.05, *p* < .0001 (for model details, see Table 4). Accuracy trended to be higher in congruent (*M*_congruent_ = .94) compared with incongruent (*M*_incongruent_ = .93), *t*(83) = 1.88, *p* = .063, trials.

**Table 4:** Multilevel model results for the unstandardized IAT effect in Experiment 2

Unstandardized IAT effect [s]				
Random Effects	Variance	*SD*		
Participants	0.08	0.28		
Words	0.05	0.22		
Residual	0.42	0.65		
Fixed Effects	Estimate	*SE*	|*t*|	
Intercept	1.257	0.06	23.00	
Congruency	0.068	0.01	6.05	

Congruency comprised two levels (congruent: self/verb on one key and other/noun on the other; incongruent: self/noun on one key and other/verb on the other)

A 2 × 2 exploratory ANOVA with the standardized IAT effect as the dependent variable and the key order and category matching order as the independent variables (for details on this analysis, see Experiment 1) revealed a main effect of the category matching order, *F*(1, 80) = 5.0, *p* = .004, η_G_^2^ = .10. That is, the IAT effect was larger when verb/self and noun/other were matched first during Blocks 2 and 3 (*M*_marginal_ = .401) than when verb/other and noun/self were matched first during Blocks 2 and 3 (*M*_marginal_ = −.047). Neither the key order, *F*(1, 80) = .54, *p* = .463, η_G_^2^ = .01, nor the interaction effect, *F*(1, 80) = 1.44, *p* = .234, η_G_^2^ = .02, were significant.

#### Inclusion of Other in the Self (IOS) Scale

The IOS score did not correlate with the standardized IAT effect (*r =* .03)*, t*(82) = 0.31, *p* = .76.

#### Agency and Communion Questionnaire (ACQ)

None of the ACQ subscales (AA, AC, CM, or CW) correlated with the standardized IAT effect (all |*t*|(82) < 1.16, all *p*s > .25, all *r*s between 0 and −0.13).

#### Agency ratings

Participants rated verbs higher in agency than nouns, as indicated by a two-sided dependent *t* test (*M*_Δ_ = 0.37), *t*(83) = 4.90, *p* < .001.

### Discussion

Results from Experiment 2 confirm our hypothesis: The finding from Experiment 1 that self (vs. other) pronouns were more closely related to verbs than to nouns was replicated. Similar to Experiment 1, exploratory analyses did not support the notion that the IAT effect is diminished when the concept of the other overlaps with the self-concept. They also do not suggest that the IAT effect scales with trait agency or communion.

## General discussion

In the present manuscript, the IAT effects found in two experiments with different samples and stimuli consistently indicated that words referring to the self (e.g., “me” or “my”) are more closely related to verbs in comparison to nouns than words referring to another person (e.g., “his” or “him”) are. The finding confirms major assumptions from theoretical models of agency and social cognition that predict a link between agency and verbs (e.g., Fausey & Boroditsky, 2011; Formanowicz et al., 2017) and between self and agency (e.g., Abele & Wojciszke, 2007, 2014). Our results suggest that the high relevance of verbs (e.g., Formanowicz, 2020; Formanowicz et al., 2017; Vigliocco et al., 2011) and agency-related linguistic expressions (Abele & Wojciszke, 2007, 2014) might be partially due to their ties to the self.

### Potential alternative explanations

An important alternative explanation for the pattern observed in the presented research is that the verb–self link, as indicated by the IAT effect, is driven by increased contextual similarity between verbs or nouns and pronouns usage. Indeed, in Experiment 1, we observed that in natural language, self-related pronouns had lower contextual similarity with respect to nouns than other-related pronouns (see SOM for details). For Experiment 2, however, we excluded this possibility as self-related (vs. other-related) pronouns and experimental stimuli were occurring in similar contexts with respect to nouns and verbs. To examine a more general usage pattern of verbs and nouns in relation to pronouns we additionally conducted a second analysis presented in the SOM in which we applied a masked language model to predict words in the context of the pronouns that were used in both experiments (Devlin et al., 2019). Importantly, general patterns of natural language use indicate that there is no privileged occurrence of verbs in relation to the self-related pronouns and that for some pronouns we detect minor differences in nouns usage in a way that self-related pronouns are more likely to occur with nouns. However, this similarity should facilitate combined processing of self-related pronouns and nouns and as a result decrease the observed IAT effect. Therefore, we deem this alternative explanation unlikely.

Furthermore, we want to note that even though we did not detect any systematic differences between natural language cooccurrence of self-related (vs. other-related) pronouns and verbs and nouns, the existence of such pattern would not speak against the hypothesis examined in this research. The privileged processing of agentic cues and of cues related to one’s agency is evident in studies of infant cognition, suggesting that humans, even before they have acquired semantic or syntactic structures of any language, represent events in terms of categories of agent and patient (for a review, see Csibra & Gergely, 2007; Kelso, 2016; Rissman & Majid, 2019; Strickland, 2017). In this context, the cognition of agency can be viewed as more fundamental than any cognition involving language. Accordingly, the primary source of the verb–self link might not be situated in language but might rather sit at the very core of human cognitive abilities.

Another potential alternative explanation of the present results is that concreteness rather than agency contributes to the self–verb link. The linguistic category model (LCM) and its extensions (Carnaghi et al., 2008; Semin & Fiedler, 1988) posit that grammatical categories vary in terms of abstractness. Verbs are seen as the most concrete and related to a specific behavior, whereas nouns are the most abstract and pertain to a person’s traits or dispositions rather than behaviors. Similarly, according to construal level theory, actions can be processed at two different levels: (1) concrete, which is related to psychologically near events conceptualized through how actions should be performed, and (2) abstract, which is related to psychologically distant events conceptualized in terms of a globalized action purpose (Trope & Liberman, 2003). According to the latter account, self could be seen as something psychologically close, encompassing high concreteness, and would therefore match verbs that are also concrete according to LCM. Following this rationale, concreteness, rather than agency, would be responsible for the IAT results. We cannot rule out this possibility entirely, especially when considering that there is a relationship between agency and concreteness (Palmeira, 2015) in the way that those who speak in a concrete way are also seen as more agentic. Note, however, that, in terms of grammatical categories, this was not evident in pseudoword perception (Formanowicz et al., 2017). That is, pseudoverbs and pseudonouns were seen as similarly abstract, although their agency ratings varied. This indicates that the grammatical category, per se, does not convey abstract references but is rather tied to the actual meaning of a word. When construing our stimuli set, we took care that both verbs and nouns matched regarding evaluated concreteness; therefore, the alternative explanation of concreteness being responsible for the obtained results pattern seems unlikely.

### Limitations

It is puzzling why the IAT effect in both experiments was larger when congruent (i.e., verb/self and noun/other response key assignments) blocks appeared before rather than after incongruent (i.e., verb/other and noun/self) ones. Such order effects have been observed before (e.g., Greenwald et al., 1998, 2003; Nosek et al., 2005) and are commonly of substantial magnitude (e.g., ~129 ms in the incongruent block, first condition, in comparison to ~224 ms in the congruent block, first condition; Greenwald et al., 1998). Order effects have been termed an undesired methodological artifact (Greenwald et al., 2009) and are not yet fully understood. A promising explanation, however, refers to task set inertia (Klauer & Mierke, 2005). In short, when the congruent block is presented first, participants do not need to inhibit any of the two categorization task sets (i.e., using the verb/noun set or the self/other set leads to the same action, thus simplifying the task), thereby laying the foundation of the IAT effect. However, when the incongruent block is presented first, participants do need to inhibit irrelevant task sets in a stimulus-dependent manner. Crucially, such inhibition has been shown to outlast the incongruent block and spill over to the congruent block, thus diminishing performance in the congruent block and therefore explaining unwanted IAT order effects (Klauer & Mierke, 2005). Please note that, following that rationale, the diminishment would have been so substantial in the present study that it would have completely overshadowed any IAT effect in the incongruent block. However, given the comparably low effect size of the present study (*d* = .25 in comparison to *d* > 1 in the original IAT study; Greenwald et al., 1998), such overshadowing seems plausible. One suggestion to reduce the order effect in future studies would be to increase practice trials so that more time passes after the incongruent block to reduce inhibition aftereffects. However, in the present data, an unreported exploratory analysis revealed that the order effect persisted even when only using the second half of the combined trials (second half of Blocks 2 and 4 in Fig. 1a) for analysis. Following the practice rationale, task set inhibition should have been diminished already. Thus, the inhibition effect might be too robust to be overcome by practice trials.

As mentioned before, the presently reported effect size (*d* = .25) is rather small in comparison to effect sizes larger than 1 in the previous IAT studies (Greenwald et al., 1998). In that study, large effect sizes were, for example, obtained by pairing pleasant/unpleasant words with flower or insect names or by stimuli targeting ethnic discrimination. In other words, the association between a flower and pleasantness is likely higher than the association between a verb and the self. This should be kept in mind when interpreting our results. However, given the narrowness of, for example, flowers’ names as stimuli in comparison to the broadness of our stimuli that are supposed to represent the entirety of verbs, the smaller effect size might come as no surprise. While counterexamples exist, IAT effects of .25 to .30 have been frequently reported for word-based IATs, with picture-based IATs leading to smaller effect sizes around .15 (Foroni & Bel-Bahar, 2010).

Lastly, please note that the present study only used male-related stimuli like “he” and “his” to represent the other (vs. self) category. This is an obvious limitation of our study However, this choice of other-related stimuli likely puts our results at the more conservative end. Research on gender stereotyping (including studies on language) indicate that male targets are seen as particularly agentic (Formanowicz & Hansen, 2022). In that respect, male agents are thus more similar to the self than female agents. Hence, we expect the IAT effect to increase if female-related, and thus less agentic pronouns like “she” or “her” would be used instead of their male-related counterparts. Also note that an explorative ANOVA for the standardized IAT effect with the participants’ gender as a factor indicated no significant differences in how women and men responded to the presented stimuli.^6^

### Future directions

The IAT is known to be a valid predictor of human behavior and, due to its implicit nature, is likely more valid than self-reports in domains with extensive impression management (Greenwald et al., 2009), such as the domain of the self. That being said, it is clear that the reported IAT effects are only a starting point for exploring the nature of the self–verb link and the associated triad. So where do the present findings leave us? From the authors’ perspective, it leaves us at a place with great potential. Could deliberate verb use be a window to the recipient’s self just as linguistic self-reference boosts emotional valence and evaluation speed (Meixner & Herbert, 2018; Weis & Herbert, 2017) and cortical/neural responses (Herbert et al., 2011)? Do people deliberately prefer verb-based expressions when talking about themselves? Does the coupled use of self-related pronouns and verbs stimulate particularly deep neural processing given that linguistic self-reference is already associated with increased activation of neural regions responsible for processing somatic and visceral sensations (e.g., Esslen et al., 2008)? These questions provide a highly rewarding path to take for future endeavors. The observed linguistic verb–agency link adds to our understanding of how the high behavioral relevance of agency in social cognition (Abele & Wojciszke, 2007, 2014) can be implemented in everyday verbal communication and opens up pathways to understanding emotional processing (which is known to be modulated by linguistic self-reference; e.g., Herbert et al., 2011; Meixner & Herbert, 2018; Weis & Herbert, 2017) of agency-related expressions. Because self-relatedness is known to boost emotional processing (e.g., Herbert et al., 2011; Meixner & Herbert, 2018; Weis & Herbert, 2017), the verb–self link also provides grounds for future promising insights into the interrelatedness of agency and emotions.

## Footnotes

1 https://CRAN.R-project.org/package=emmeans

2 The associated R analysis script and the data sets generated and analyzed during the current study are available in the Open Science Framework (https://osf.io/q8zkp/).

3 *t*^2^ *= F,* thus *F* = 1.48.

4 The associated R analysis script and the data sets generated and analyzed during the current study are available in the Open Science Framework (https://osf.io/q8zkp/).

5 *t*^2^ *= F*, thus *F* = 1.51.

6 Experiment 1: *F*(1, 81) = 1.9, *p* = .169, *M*_male_ = .21, *M*_female_ = .04, 1 participant preferred not to disclose gender and was excluded from this analysis; Experiment 2: *F*(1, 81) = 1.0, *p* = .322, *M*_male_ = .28, *M*_female_ = .11, 1 participant identified as diverse and was excluded from this analysis.
